# An Experience of Being a Patient as a Medical Student

**DOI:** 10.31729/jnma.7283

**Published:** 2022-05-31

**Authors:** Krisha Shakya

**Affiliations:** 1 KIST Medical College and Teaching Hospital, Imadol, Lalitpur, Nepal

**Keywords:** *empathy*, *hemoptysis*, *medical students*

## Abstract

"Walk a mile in someone else's shoes" is a reminder to practice empathy. Reading about the diseases in a textbook but encountering that disease is a nightmare. This article highlights the feeling of a medical student being a patient and learning from the experience. Learning from the experience will always be deeply seated in the person. It provides readers with a better understanding to practice empathy as a medical student and knowledge of the treatment of hemoptysis.

## INTRODUCTION

Hemoptysis is the expectoration of blood from the lung parenchyma or airways. The initial step in the evaluation is determining the origin of the bleeding.^1^ Most cases of hemoptysis are due to lung infections, bronchiectasis, lung cancer and pulmonary vascular disorders. The extent of haemorrhage is decisive for the choice of investigational and therapeutic measures. Massive hemoptysis is always life-threatening and requires immediate action.^2^ Bronchial artery embolization, rigid and flexible bronchoscopy, and surgery all serve as potential treatment options apart from tranexamic acid to provide definitive control of hemorrhage.^3^ Medical students theoretically learn about empathy and doctor-patient relationship but without walking in someone else's shoes we do not truly understand the meaning.

## EXPERIENCE

'Hemoptysis' is the chapter we medical students learn. However, the scenario is different when being rushed to the hospital because of hemoptysis and the mind of a medical student surfing through the main causes of hemoptysis. The experience of a medical student being a patient is kind of a learning process. We get to observe in detail how the doctors, nurses and other medical staff work as a team without breaking the chain of passing on correct information.

The thought of working in the Intensive Care Unit (ICU) for medical students in the future is like a dream come true for gaining good experience but from the perspective of a medical student being admitted to the ICU is the worst nightmare. The sound of physiological monitors beeping and the unstable patients in the ICU is a horrifying situation. We never learn to work on understanding patients' feelings in our journey as a medical student. Rather we take each patient and deal with it as a case. When we end up in the place of a patient, instead of assurance we are told to take the procedures presented to us as a practical lesson. This is where you begin to realize the importance of empathy and compassion as a medical student.

## LEARNING FROM THE SURROUNDING

Although in practical classes, medical students role-play being a patient and doctor to sharpen their practical skills, in reality, the scenario is different. While students may understand the importance of empathy, there is currently no consensus on the appropriate method for teaching this quality.^4^ Being a patient and from that view everything is different. Sometimes getting frustrated with the procedures or with the system of the hospital. They would lose their patience and temper and get frustrated by getting confined within the four walls of the room in the hospital bed. To know they are receiving the very best care and that is only conveyed when the team is empathetic. After days of hospital stay, I learnt that when there is improved two-way communication, a better treatment is achieved. Empathy works when it goes both ways. Recently, doctors and nurses have been overburdened with work and even their efforts have not been reciprocated. However, the patient's party should show respect, and have patience and faith in the process.

Hospitalization teaches you the importance of family support and friends. Family and friends could instantly uplift your morale and somehow play a role in your recovery process.

Inhaled and intravenous Tranexamic Acid (TXA) has always been the choice of drug for hemoptysis as it is an anti-fibrinolytic agent and it helped a lot to control my hemoptysis. According to the presented evidence, TXA use may afford successful treatment of hemoptysis because it can reduce further intervention risk and shorten the bleeding duration and length of hospital stay.^5^ In hemoptysis, after excluding extrapulmonary sites of bleeding, computed tomography and bronchoscopy are complementary and may indicate pathologies not detectable by chest radiograph. An interdisciplinary approach including pneumologist, thoracic surgeon, Ear Nose Throat (ENT) and radiologist in the management of hemoptysis is essential.^6^ The hemoptysis treatment approach is vast but the increase in research and medical studies has led to prompt diagnosis and treatment.

The entire experience of being a patient in the hospital as a medical student teaches you where you are lacking in humanism. Doctors must understand the expectations patients have during their visits to the hospital. Empathy is more than just diagnosis and treatment. When we take each patient as a case in our journey of medical school we never learn to deal with patients which in turn leads to patients' lack of trust regarding the treatment being received. When patients perceive that they actually connect on common ground with the medical team and their emotions are being acknowledged, they have a better recovery. However, we have a long way to go before incorporating empathy into our medical practice.

## WAY FORWARD

Being in the shoes of a patient makes a medical student realize that it all comes down to empathy. Sometimes the cases presented to us in clinical settings might differ from the chapters we learn, however, we must be ready to tackle the problem. Those students who have had higher-level formal training in the development of empathy are able to attend to the patient holistically, rather than on a purely medical basis. The experience of being a patient made a medical student realize how necessary it is for doctors to recognize and validate a patient's fear, anxiety and worry. Medical life is not confined to books and clinical practice but it is also about practising humanism. Empathy is a topic in the curriculum which is undervalued by the students. Treat patients like you treat yourself.
